# 2-{3-Cyano-5,5-dimethyl-4-[6-(pyrrol­i­din-1-yl)hexa-1,3,5-trien­yl]-2,5-dihydro-2-furylidene}malononitrile

**DOI:** 10.1107/S1600536808028110

**Published:** 2008-09-27

**Authors:** Graeme J. Gainsford, M. Delower H. Bhuiyan, Andrew J. Kay

**Affiliations:** a Industrial Research Limited, PO Box 31-310, Lower Hutt, New Zealand

## Abstract

The title compound, C_20_H_20_N_4_O, is packed into a three-dimensional ‘herringbone’ matrix using two different types of attractive C—H⋯N(cyano) inter­actions. The bond-length alternation, caused by delocalization of charge between the donor N atoms and the cyano acceptor groups, is compared with related compounds.

## Related literature

For general background, see: Kay *et al.* (2004 ▶). For related literature, see: Gainsford *et al.* (2007 ▶, 2008*a* ▶,*b* ▶); Marder *et al.* (1993 ▶); Li *et al.* (2005 ▶). For a similar herringbone structure, see: Desiraju & Gavezzotti (1989 ▶).
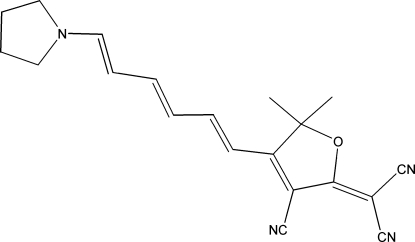


## Experimental

### 

#### Crystal data

C_20_H_20_N_4_O
*M* *_r_* = 332.40
Orthorhombic, 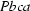

*a* = 12.6766 (13) Å
*b* = 11.7603 (13) Å
*c* = 24.164 (3) Å
*V* = 3602.4 (7) Å^3^
*Z* = 8
Mo *K*α radiation
μ = 0.08 mm^−1^
*T* = 116 (2) K
0.32 × 0.25 × 0.07 mm

#### Data collection

Bruker Nonius APEXII CCD area-detector diffractometer
Absorption correction: multi-scan (*SADABS*; Sheldrick, 1996 ▶) *T*_min_ = 0.494, *T*_max_ = 1.0 (expected range = 0.491–0.995)
20272 measured reflections
3641 independent reflections
2078 reflections with *I* > 2σ(*I*)
*R*_int_ = 0.081

#### Refinement

*R*[*F* ^2^ > 2σ(*F* ^2^)] = 0.048
*wR*(*F* ^2^) = 0.129
*S* = 0.98
3641 reflections
229 parameters
H-atom parameters constrained
Δρ_max_ = 0.26 e Å^−3^
Δρ_min_ = −0.27 e Å^−3^

### 

Data collection: *APEX2* (Bruker, 2005 ▶); cell refinement: *APEX2*; data reduction: *SAINT* (Bruker, 2005 ▶); program(s) used to solve structure: *SHELXS97* (Sheldrick, 2008 ▶); program(s) used to refine structure: *SHELXL97* (Sheldrick, 2008 ▶); molecular graphics: *ORTEP-3* (Farrugia, 1997 ▶) and *PLATON* (Spek, 2003 ▶); software used to prepare material for publication: *SHELXL97*, *PLATON* and *Mercury* (Macrae *et al.*, 2006 ▶).

## Supplementary Material

Crystal structure: contains datablocks global, I. DOI: 10.1107/S1600536808028110/ng2475sup1.cif

Structure factors: contains datablocks I. DOI: 10.1107/S1600536808028110/ng2475Isup2.hkl

Additional supplementary materials:  crystallographic information; 3D view; checkCIF report

## Figures and Tables

**Table 1:** Hydrogen-bond geometry (Å, °)

*D*—H⋯*A*	*D*—H	H⋯*A*	*D*⋯*A*	*D*—H⋯*A*
C12—H12⋯N3^i^	0.95	2.49	3.379 (3)	156
C17—H17*A*⋯N1^ii^	0.99	2.61	3.402 (3)	137
C20—H20*B*⋯N2^ii^	0.99	2.56	3.316 (3)	133

Symmetry codes: (i) 
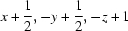
; (ii) 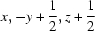.

**Table 2:** Selected bond lengths and angles (Å,°) in the title compound and related compounds Compound (I) is the title compound, (II) is the closely related buta-1,3-dienyl equivalent (Gainsford *et al.*, 2008*c*), (III) is the piperidin-1-yl equivalent of (II) (Gainsford *et al.*, 2008*b* ▶) and (IV) is the starting acceptor molecule 2-dicyanomethylene-4,5,5-trimethyl-2,5-dihydro-furan-3-carbonitrile (Li *et al.*, 2005 ▶)

	(I)	(II)^*a*^	(III)^*b*^	(IV)
C2—C6	1.388 (3)	1.388 (5)	1.389 (6)	1.359 (4)
C6—C7	1.401 (3)	1.412 (5)	1.390 (5)	1.445 (4)
C4—C7	1.417 (3)	1.402 (5)	1.426 (6)	1.343 (4)
C4—C11	1.374 (3)	1.405 (5)	1.366 (5)	1.472 (4)
C11—C12	1.410 (3)	1.397 (5)	1.402 (6)	—
C12—C13	1.369 (3)	1.384 (5)	1.381 (5)	—
C13—C14	1.407 (3)	1.397 (5)	1.396 (6)	—
C14—C15	1.369 (3)	—	—	—
C15—C16	1.404 (3)	—	—	—
N4—CT^*c*^	1.306 (2)	1.317 (4)	1.315 (5)	—
C6—O1	1.351 (2)	1.341 (4)	1.360 (4)	1.333 (3)
C5—O1	1.486 (2)	1.482 (4)	1.489 (8)	1.481 (4)
C4—C7—C6	109.84 (18)	108.7 (3)	109.7 (4)	109.4 (2)
C7—C6—C2	132.3 (2)	131.7 (3)	133.7 (4)	131.3 (3)
C11—C4—C7	126.6 (2)	125.2 (3)	126.6 (4)	128.6 (3)
C4—C11—C12—C13	179.6 (2)	−177.4 (4)	178.1 (4)	—

Notes: (*a*) Larger s.u. values reflect crystal quality; (*b*) Average of two independent molecules; (*c*) CT is the terminal atom of the polyene chain.

**Fig. 1.**
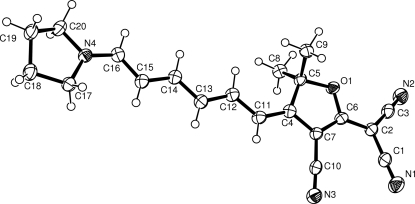
Molecular structure of the asymmetric unit (Farrugia, 1997); displacement ellipsoids are shown at the 50% probability level.

**Fig. 2.**
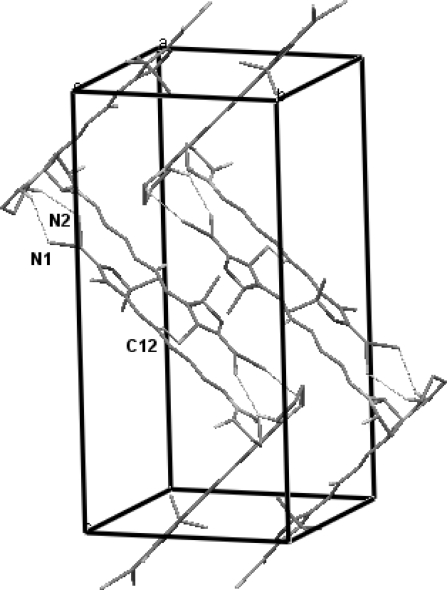
Partial packing diagram of the cell (I) (Mercury; Macrae et al., 2006) viewed approximately down the a axis. Only H atoms involved in selected C—H···N hydrogen bonds (dashed lines) are shown. Atom labels indicate the three main interactions (see text & Table 1). Colours: Nitrogen, blue; Oxygen, red; Carbon, black.

## supplementary crystallographic information

### Comment

We have previously reported on the synthesis of a number of high figure of merit chromophores for nonlinear optics (Kay *et al.*, 2004), and the X-ray crystallographic and structural properties of crucial dye precursors used [Gainsford *et al.*, 2007; Gainsford *et al.*, 2008*a* (hereafter III); Gainsford *et al.*, 2008*b* (hereafter II)]. We report here the crystallographic data of another molecule derived from a chromophore precursor and summarize the structural details of this and closely related compounds.

The asymmetric unit contents of the title compound (I) is shown in Fig. 1. The molecule possesses approximately planar geometry with a major tilt between the pendant planar dicyanomethylene groups (N1,N2,C1–C3) and the "CDFP" 5-membered ring plane (O1, C4—C7) of 11.56 (14)°. The polyene chain atoms (C4,C11–C16), which are coplanar with r.m.s. deviations of 0.026 (2) Å, make an angle of 2.07 (15)° to the "CDFP" ring plane. The pyrrolidin-1-yl ring adopts an envelope configuration with C19 the flap atom at 0.551 (2) Å out of plane (Spek, 2003).

Selected geometrical values for (I), the closely related buta-1,3-dienyl equivalent (II) (Gainsford *et al.*, 2008*c*), the piperidin-1-yl equivalent of II (III) (Gainsford *et al.*, 2008*b*) and the starting acceptor molecule 2-dicyanomethylene-4,5,5-trimethyl-2,5-dihydro-furan-3-carbonitrile (IV) (Li *et al.*, 2005) are collected in Table 2. Delocalization is apparent through the polyene chain and CDFP fragment (*e.g.* compare C4—C7 & C4—C11 bond lengths). Bond length alternation (BLA) calculations (Marder *et al.*, 1993) confirm the similarities of (I) to (II) & (III) with values of -0.027 (I), 0.005 (II) and -0.027 (III) Å compared with the value of 0.108Å in (IV). The similar values for (I) & (III) also confirm the equivalent donor strengths of the pyrolidine & piperidine N-donors.

The molecules are arranged in planes (Fig. 2) with the well known "herringbone" motif (Desiraju & Gavezzotti, 1989). The polyene C—H attractive interactions with adjacent cyano nitrogen atom N3 (principally entry 1, Table 1), commonly observed for these molecules (Gainsford *et al.*, 2008*a*), link adjacent molecules which lie parallel to each other and the 0,1,-2 plane. The other two main methylene H···N (cyano) interactions (entries 3 & 4, Table 2) link to the other (herringbone) planes which are parallel to the 0,1,2 plane.

### Experimental

To a solution of 5.8 mmole of {4-(4-Acetanilido-*trans*,*trans*-1,3,5-hexatrienyl) -3-cyano-5,5-dimethyl-2(5*H*)-furanylidene}propanedinitrile (Compound 11*c*, Kay *et al.*, 2004) in 30 ml of ethanol was added an equimolar quantity of pyrrolidine. The solution was refluxed 1 h, cooled and the product collected by filtration and washed with ethanol. λ_max_ 645 nm (pyridine); 630 nm (DMF) log_10_ε 5.11. Final crystallization was from dichloromethane.

### Refinement

All methyl and other H atoms were refined with *U*_iso_ 1.5 & 1.2 times respectively that of the *U*_eq_ of their parent atom using riding models. All non-hydrogen atoms were refined with anisotropic thermal parameters.

### Crystal data

**Table:**

C_20_H_20_N_4_O	*F*(000) = 1408
*M**_r_* = 332.40	*D*_x_ = 1.226 Mg m^−^^3^
Orthorhombic, *P**b**c**a*	Mo *K*α radiation, λ = 0.71073 Å
Hall symbol: -P 2ac 2ab	Cell parameters from 6328 reflections
*a* = 12.6766 (13) Å	θ = 2.5–25.0°
*b* = 11.7603 (13) Å	µ = 0.08 mm^−^^1^
*c* = 24.164 (3) Å	*T* = 116 K
*V* = 3602.4 (7) Å^3^	Plate, brown
*Z* = 8	0.32 × 0.25 × 0.07 mm

### Data collection

**Table:**

Bruker Nonius APEXII CCD area-detector diffractometer	3641 independent reflections
Radiation source: fine-focus sealed tube	2078 reflections with *I* > 2σ(*I*)
graphite	*R*_int_ = 0.081
Detector resolution: 8.192 pixels mm^-1^	θ_max_ = 26.4°, θ_min_ = 3.4°
φ and ω scans	*h* = −10→15
Absorption correction: multi-scan (SADABS; Sheldrick, 1996)	*k* = −14→14
*T*_min_ = 0.494, *T*_max_ = 1.0	*l* = −30→27
20272 measured reflections	

### Refinement

**Table:**

Refinement on *F*^2^	Secondary atom site location: difference Fourier map
Least-squares matrix: full	Hydrogen site location: inferred from neighbouring sites
*R*[*F*^2^ > 2σ(*F*^2^)] = 0.048	H-atom parameters constrained
*wR*(*F*^2^) = 0.129	*w* = 1/[σ^2^(*F*_o_^2^) + (0.0633*P*)^2^] where *P* = (*F*_o_^2^ + 2*F*_c_^2^)/3
*S* = 0.98	(Δ/σ)_max_ < 0.001
3641 reflections	Δρ_max_ = 0.26 e Å^−^^3^
229 parameters	Δρ_min_ = −0.27 e Å^−^^3^
0 restraints	Extinction correction: SHELXL97 (Sheldrick, 2008), Fc^*^=kFc[1+0.001xFc^2^λ^3^/sin(2θ)]^-1/4^
Primary atom site location: structure-invariant direct methods	Extinction coefficient: 0.0124 (13)

### Special details

**Table:**

Geometry. All e.s.d.'s (except the e.s.d. in the dihedral angle between two l.s. planes) are estimated using the full covariance matrix. The cell e.s.d.'s are taken into account individually in the estimation of e.s.d.'s in distances, angles and torsion angles; correlations between e.s.d.'s in cell parameters are only used when they are defined by crystal symmetry. An approximate (isotropic) treatment of cell e.s.d.'s is used for estimating e.s.d.'s involving l.s. planes.
Refinement. An extinction parameter was refined. Refinement of *F*^2^ against ALL reflections. The weighted *R*-factor *wR* and goodness of fit *S* are based on *F*^2^, conventional *R*-factors *R* are based on *F*, with *F* set to zero for negative *F*^2^. The threshold expression of *F*^2^ > σ(*F*^2^) is used only for calculating *R*-factors(gt) *etc*. and is not relevant to the choice of reflections for refinement. *R*-factors based on *F*^2^ are statistically about twice as large as those based on *F*, and *R*- factors based on ALL data will be even larger.

### Fractional atomic coordinates and isotropic or equivalent isotropic displacement parameters (Å^2^)

**Table:**

	*x*	*y*	*z*	*U*_iso_*/*U*_eq_	
O1	0.18457 (11)	0.09182 (12)	0.41943 (5)	0.0312 (4)	
N1	−0.10627 (17)	−0.09427 (19)	0.33366 (8)	0.0476 (6)	
N2	0.24246 (16)	−0.08511 (17)	0.31039 (8)	0.0447 (5)	
N3	−0.18623 (16)	0.14659 (16)	0.40666 (7)	0.0371 (5)	
N4	0.07598 (13)	0.73470 (14)	0.71061 (6)	0.0276 (4)	
C1	−0.02458 (19)	−0.05836 (19)	0.34560 (8)	0.0331 (6)	
C2	0.07610 (17)	−0.01503 (19)	0.36100 (8)	0.0289 (5)	
C3	0.16705 (19)	−0.05488 (19)	0.33319 (8)	0.0331 (6)	
C4	0.06334 (17)	0.20694 (18)	0.46776 (7)	0.0262 (5)	
C5	0.17947 (17)	0.17853 (18)	0.46411 (7)	0.0272 (5)	
C6	0.08595 (16)	0.06720 (18)	0.40190 (8)	0.0271 (5)	
C7	0.01165 (16)	0.13485 (17)	0.42942 (8)	0.0258 (5)	
C8	0.24674 (17)	0.27708 (18)	0.44398 (8)	0.0330 (5)	
H8A	0.3161	0.2487	0.4326	0.049*	
H8B	0.2554	0.3324	0.4740	0.049*	
H8C	0.2121	0.3137	0.4124	0.049*	
C9	0.22067 (17)	0.11865 (18)	0.51558 (8)	0.0321 (5)	
H9A	0.1729	0.0564	0.5255	0.048*	
H9B	0.2245	0.1730	0.5463	0.048*	
H9C	0.2912	0.0880	0.5082	0.048*	
C10	−0.09674 (19)	0.13888 (18)	0.41677 (8)	0.0274 (5)	
C11	0.01687 (17)	0.28779 (18)	0.50081 (8)	0.0294 (5)	
H11	−0.0572	0.2970	0.4971	0.035*	
C12	0.06770 (18)	0.35851 (18)	0.53967 (8)	0.0292 (5)	
H12	0.1418	0.3493	0.5437	0.035*	
C13	0.02036 (17)	0.43921 (18)	0.57211 (8)	0.0290 (5)	
H13	−0.0539	0.4490	0.5698	0.035*	
C14	0.07818 (17)	0.50812 (18)	0.60878 (7)	0.0277 (5)	
H14	0.1523	0.4964	0.6100	0.033*	
C15	0.03820 (17)	0.59043 (18)	0.64288 (8)	0.0284 (5)	
H15	−0.0358	0.6031	0.6443	0.034*	
C16	0.10612 (18)	0.65628 (18)	0.67577 (8)	0.0282 (5)	
H16	0.1797	0.6427	0.6724	0.034*	
C17	−0.03553 (16)	0.75976 (18)	0.72429 (8)	0.0305 (5)	
H17A	−0.0717	0.6910	0.7383	0.037*	
H17B	−0.0739	0.7882	0.6914	0.037*	
C18	−0.02940 (18)	0.85123 (19)	0.76908 (9)	0.0363 (6)	
H18A	−0.0828	0.8376	0.7982	0.044*	
H18B	−0.0410	0.9277	0.7531	0.044*	
C19	0.08212 (17)	0.84082 (19)	0.79260 (8)	0.0358 (6)	
H19A	0.1061	0.9139	0.8086	0.043*	
H19B	0.0855	0.7813	0.8215	0.043*	
C20	0.14812 (18)	0.80835 (18)	0.74275 (8)	0.0340 (6)	
H20A	0.1689	0.8764	0.7212	0.041*	
H20B	0.2124	0.7665	0.7540	0.041*	

### Atomic displacement parameters (Å^2^)

**Table:**

	*U*^11^	*U*^22^	*U*^33^	*U*^12^	*U*^13^	*U*^23^
O1	0.0281 (9)	0.0392 (9)	0.0264 (7)	0.0026 (7)	−0.0026 (6)	−0.0084 (6)
N1	0.0432 (14)	0.0531 (14)	0.0466 (12)	−0.0001 (11)	−0.0019 (10)	−0.0186 (11)
N2	0.0464 (14)	0.0491 (13)	0.0385 (11)	0.0012 (11)	0.0056 (10)	−0.0111 (10)
N3	0.0351 (13)	0.0421 (13)	0.0342 (10)	−0.0013 (9)	0.0022 (9)	−0.0045 (9)
N4	0.0303 (11)	0.0320 (10)	0.0205 (8)	−0.0010 (8)	0.0004 (7)	−0.0002 (8)
C1	0.0395 (15)	0.0351 (14)	0.0248 (11)	0.0029 (11)	−0.0006 (10)	−0.0078 (10)
C2	0.0336 (13)	0.0321 (13)	0.0209 (10)	0.0020 (10)	−0.0007 (9)	−0.0024 (9)
C3	0.0420 (16)	0.0339 (13)	0.0235 (11)	−0.0008 (11)	−0.0009 (10)	−0.0051 (10)
C4	0.0301 (13)	0.0298 (12)	0.0189 (10)	−0.0005 (10)	0.0019 (9)	0.0018 (9)
C5	0.0296 (13)	0.0311 (12)	0.0209 (10)	−0.0003 (10)	−0.0004 (9)	−0.0043 (9)
C6	0.0302 (13)	0.0322 (13)	0.0190 (10)	−0.0019 (10)	−0.0025 (9)	0.0019 (9)
C7	0.0266 (13)	0.0306 (13)	0.0201 (10)	0.0006 (9)	−0.0002 (9)	−0.0015 (9)
C8	0.0336 (13)	0.0380 (13)	0.0272 (11)	−0.0014 (11)	0.0024 (10)	0.0029 (10)
C9	0.0370 (14)	0.0325 (13)	0.0267 (11)	0.0030 (10)	−0.0020 (10)	0.0019 (10)
C10	0.0343 (15)	0.0283 (13)	0.0197 (10)	−0.0018 (10)	0.0042 (10)	−0.0035 (9)
C11	0.0291 (13)	0.0382 (14)	0.0211 (10)	0.0005 (10)	−0.0004 (9)	−0.0017 (10)
C12	0.0308 (13)	0.0356 (13)	0.0212 (10)	−0.0004 (10)	0.0017 (9)	0.0004 (9)
C13	0.0328 (13)	0.0339 (13)	0.0204 (10)	0.0013 (10)	0.0011 (9)	−0.0007 (9)
C14	0.0337 (13)	0.0308 (12)	0.0186 (10)	−0.0008 (10)	0.0022 (9)	0.0030 (9)
C15	0.0336 (13)	0.0327 (13)	0.0190 (10)	0.0002 (10)	0.0015 (9)	0.0008 (9)
C16	0.0341 (13)	0.0303 (12)	0.0200 (10)	0.0023 (10)	0.0048 (9)	0.0029 (9)
C17	0.0330 (14)	0.0359 (13)	0.0225 (10)	0.0031 (10)	0.0002 (9)	−0.0008 (9)
C18	0.0490 (16)	0.0341 (13)	0.0258 (11)	0.0033 (11)	−0.0010 (11)	−0.0045 (10)
C19	0.0451 (16)	0.0352 (13)	0.0270 (11)	0.0002 (11)	−0.0043 (10)	−0.0071 (10)
C20	0.0440 (15)	0.0324 (13)	0.0257 (11)	−0.0062 (11)	−0.0058 (10)	−0.0020 (10)

### Geometric parameters (Å, °)

**Table:**

O1—C6	1.351 (2)	C9—H9C	0.9800
O1—C5	1.486 (2)	C11—C12	1.410 (3)
N1—C1	1.155 (3)	C11—H11	0.9500
N2—C3	1.159 (3)	C12—C13	1.369 (3)
N3—C10	1.164 (3)	C12—H12	0.9500
N4—C16	1.306 (2)	C13—C14	1.407 (3)
N4—C20	1.480 (3)	C13—H13	0.9500
N4—C17	1.481 (3)	C14—C15	1.369 (3)
C1—C2	1.424 (3)	C14—H14	0.9500
C2—C6	1.388 (3)	C15—C16	1.404 (3)
C2—C3	1.414 (3)	C15—H15	0.9500
C4—C11	1.374 (3)	C16—H16	0.9500
C4—C7	1.417 (3)	C17—C18	1.528 (3)
C4—C5	1.512 (3)	C17—H17A	0.9900
C5—C8	1.519 (3)	C17—H17B	0.9900
C5—C9	1.522 (3)	C18—C19	1.529 (3)
C6—C7	1.401 (3)	C18—H18A	0.9900
C7—C10	1.408 (3)	C18—H18B	0.9900
C8—H8A	0.9800	C19—C20	1.516 (3)
C8—H8B	0.9800	C19—H19A	0.9900
C8—H8C	0.9800	C19—H19B	0.9900
C9—H9A	0.9800	C20—H20A	0.9900
C9—H9B	0.9800	C20—H20B	0.9900
			
C6—O1—C5	109.54 (15)	C13—C12—C11	126.1 (2)
C16—N4—C20	124.81 (18)	C13—C12—H12	116.9
C16—N4—C17	124.30 (17)	C11—C12—H12	116.9
C20—N4—C17	110.87 (16)	C12—C13—C14	122.1 (2)
N1—C1—C2	179.2 (2)	C12—C13—H13	118.9
C6—C2—C3	119.7 (2)	C14—C13—H13	118.9
C6—C2—C1	121.06 (19)	C15—C14—C13	126.4 (2)
C3—C2—C1	119.22 (19)	C15—C14—H14	116.8
N2—C3—C2	178.5 (2)	C13—C14—H14	116.8
C11—C4—C7	126.6 (2)	C14—C15—C16	120.2 (2)
C11—C4—C5	127.16 (18)	C14—C15—H15	119.9
C7—C4—C5	106.25 (17)	C16—C15—H15	119.9
O1—C5—C4	103.67 (15)	N4—C16—C15	125.1 (2)
O1—C5—C8	105.46 (15)	N4—C16—H16	117.5
C4—C5—C8	113.37 (17)	C15—C16—H16	117.5
O1—C5—C9	105.15 (16)	N4—C17—C18	104.45 (16)
C4—C5—C9	112.87 (16)	N4—C17—H17A	110.9
C8—C5—C9	114.97 (17)	C18—C17—H17A	110.9
O1—C6—C2	117.11 (18)	N4—C17—H17B	110.9
O1—C6—C7	110.58 (17)	C18—C17—H17B	110.9
C2—C6—C7	132.3 (2)	H17A—C17—H17B	108.9
C6—C7—C10	124.89 (18)	C17—C18—C19	104.71 (17)
C6—C7—C4	109.84 (18)	C17—C18—H18A	110.8
C10—C7—C4	124.96 (18)	C19—C18—H18A	110.8
C5—C8—H8A	109.5	C17—C18—H18B	110.8
C5—C8—H8B	109.5	C19—C18—H18B	110.8
H8A—C8—H8B	109.5	H18A—C18—H18B	108.9
C5—C8—H8C	109.5	C20—C19—C18	103.61 (17)
H8A—C8—H8C	109.5	C20—C19—H19A	111.0
H8B—C8—H8C	109.5	C18—C19—H19A	111.0
C5—C9—H9A	109.5	C20—C19—H19B	111.0
C5—C9—H9B	109.5	C18—C19—H19B	111.0
H9A—C9—H9B	109.5	H19A—C19—H19B	109.0
C5—C9—H9C	109.5	N4—C20—C19	102.91 (17)
H9A—C9—H9C	109.5	N4—C20—H20A	111.2
H9B—C9—H9C	109.5	C19—C20—H20A	111.2
N3—C10—C7	177.4 (2)	N4—C20—H20B	111.2
C4—C11—C12	126.8 (2)	C19—C20—H20B	111.2
C4—C11—H11	116.6	H20A—C20—H20B	109.1
C12—C11—H11	116.6		
			
C6—O1—C5—C4	−3.51 (19)	C5—C4—C7—C6	−1.4 (2)
C6—O1—C5—C8	−122.90 (17)	C11—C4—C7—C10	3.6 (3)
C6—O1—C5—C9	115.19 (17)	C5—C4—C7—C10	−175.33 (18)
C11—C4—C5—O1	−176.05 (18)	C7—C4—C11—C12	178.9 (2)
C7—C4—C5—O1	2.9 (2)	C5—C4—C11—C12	−2.4 (3)
C11—C4—C5—C8	−62.2 (3)	C4—C11—C12—C13	179.6 (2)
C7—C4—C5—C8	116.70 (18)	C11—C12—C13—C14	−178.23 (19)
C11—C4—C5—C9	70.7 (3)	C12—C13—C14—C15	−179.8 (2)
C7—C4—C5—C9	−110.35 (18)	C13—C14—C15—C16	−177.29 (19)
C5—O1—C6—C2	−178.33 (17)	C20—N4—C16—C15	−176.28 (19)
C5—O1—C6—C7	2.8 (2)	C17—N4—C16—C15	5.4 (3)
C3—C2—C6—O1	−10.6 (3)	C14—C15—C16—N4	−178.18 (19)
C1—C2—C6—O1	170.85 (18)	C16—N4—C17—C18	176.39 (18)
C3—C2—C6—C7	168.0 (2)	C20—N4—C17—C18	−2.2 (2)
C1—C2—C6—C7	−10.6 (3)	N4—C17—C18—C19	−20.2 (2)
O1—C6—C7—C10	173.07 (18)	C17—C18—C19—C20	34.8 (2)
C2—C6—C7—C10	−5.5 (4)	C16—N4—C20—C19	−154.84 (18)
O1—C6—C7—C4	−0.9 (2)	C17—N4—C20—C19	23.7 (2)
C2—C6—C7—C4	−179.5 (2)	C18—C19—C20—N4	−35.4 (2)
C11—C4—C7—C6	177.55 (19)		

### Hydrogen-bond geometry (Å, °)

**Table:**

*D*—H···*A*	*D*—H	H···*A*	*D*···*A*	*D*—H···*A*
C12—H12···N3^i^	0.95	2.49	3.379 (3)	156
C17—H17A···N1^ii^	0.99	2.61	3.402 (3)	137
C20—H20B···N2^ii^	0.99	2.56	3.316 (3)	133

Symmetry codes: (i) *x*+1/2, −*y*+1/2, −*z*+1; (ii) *x*, −*y*+1/2, *z*+1/2.

### Table 2 Selected bond lengths and angles (Å,°) in the title compound and related compounds

Compound (I) is the title compound, (II) is the closely related buta-1,3-dienyl equivalent (Gainsford *et al.*, 2008*c*), (III) is the piperidin-1-yl equivalent of (II) (Gainsford *et al.*, 2008*b*) and (IV) is the starting acceptor molecule 2-dicyanomethylene-4,5,5-trimethyl-2,5-dihydro-furan-3-carbonitrile (Li *et al.*, 2005)

**Table:**

?s	(I)	(II)^a^	(III)^b^	(IV)
C2—C6	1.388 (3)	1.388 (5)	1.389 (6)	1.359 (4)
C6—C7	1.401 (3)	1.412 (5)	1.390 (5)	1.445 (4)
C4—C7	1.417 (3)	1.402 (5)	1.426 (6)	1.343 (4)
C4—C11	1.374 (3)	1.405 (5)	1.366 (5)	1.472 (4)
C11—C12	1.410 (3)	1.397 (5)	1.402 (6)	—
C12—C13	1.369 (3)	1.384 (5)	1.381 (5)	—
C13—C14	1.407 (3)	1.397 (5)	1.396 (6)	—
C14—C15	1.369 (3)	—	—	—
C15—C16	1.404 (3)	—	—	—
N4—CT^c^	1.306 (2)	1.317 (4)	1.315 (5)	—
C6—O1	1.351 (2)	1.341 (4)	1.360 (4)	1.333 (3)
C5—O1	1.486 (2)	1.482 (4)	1.489 (8)	1.481 (4)
C4—C7—C6	109.84 (18)	108.7 (3)	109.7 (4)	109.4 (2)
C7—C6—C2	132.3 (2)	131.7 (3)	133.7 (4)	131.3 (3)
C11—C4—C7	126.6 (2)	125.2 (3)	126.6 (4)	128.6 (3)
C4—C11—C12—C13	179.6 (2)	-177.4 (4)	178.1 (4)	—

Notes: (*a*) Larger s.u. values reflect crystal quality; (*b*) Average of two independent molecules; (*c*) CT is the terminal atom of the polyene chain.
